# Magnetic fluid hyperthermia inhibits the growth of breast carcinoma and downregulates vascular endothelial growth factor expression

**DOI:** 10.3892/ol.2014.1893

**Published:** 2014-02-20

**Authors:** GUIHUA WANG, DERONG XU, QIN CHAI, XIAOLANG TAN, YU ZHANG, NING GU, JINTIAN TANG

**Affiliations:** 1 Cancer Center/Cancer Research Institute, Changsha Central Hospital, Changsha, Hunan 410004, P.R. China; 2 Cancer Center, Xiangya Hospital, Central South University, Changsha, Hunan 410008, P.R. China; 3 Laboratory for Jiangsu Biomaterials and Devices, State Key Laboratory of Bioelectronics, Southeast University, Nanjing, Jiangsu 210096, P.R. China; 4 Medical Physics and Engineering Institute, Department of Engineering Physics, Tsinghua University, Beijing 100084, P.R. China

**Keywords:** breast carcinoma, thermotherapy, magnetic nanoparticle, vascular endothelial growth factor, angiogenesis

## Abstract

The application of magnetic fluid hyperthermia (MFH) with nanoparticles has been shown to inhibit tumor growth in several animal models. However, the feasibility of using MFH *in vivo* to treat breast cancer is uncertain, and the mechanism is unclear. In the present study, it was observed that the intratumoral administration of MFH induced hyperthermia significantly in rats with Walker-265 breast carcinomas. The hyperthermia treatment with magnetic nanoparticles inhibited tumor growth *in vivo* and promoted the survival of the tumor-bearing rats. Furthermore, it was found that MFH treatment downregulated the protein expression of vascular endothelial growth factor (VEGF) in the tumor tissue, as observed by immunohistochemistry. MFH treatment also decreased the gene expression of VEGF and its receptors, VEGF receptor 1 and 2, and inhibited angiogenesis in the tumor tissues. Taken together, these results indicate that the application of MFH with nanoparticles is feasible for the treatment of breast carcinoma. The MFH-induced downregulation of angiogenesis may also contribute to the induction of an anti-tumor effect.

## Introduction

Hyperthermia is a promising approach for cancer therapy. Various methods, including the use of hot water, capacitive heating and magnetic nanoparticles with an alternating magnetic field, have been reported to induce hyperthermia (1). Magnetic nanoparticles generate heat under an alternating magnetic field by hysteresis loss. The advantages of magnetic particles for hyperthermia induction are biocompatibility, injectability, lack of toxicity, effective energy absorption of the alternating magnetic field and high-level accumulation in the target tumor (2). Typically, there are 2 ranges of targeting temperature used in hyperthermic treatment. High temperatures are ≥50°C and low temperatures are from 40–43°C (2). High temperatures are usually supposed to kill targeted tissue directly (3–5). However, focal hyperthermia has been reported to induce an anti-tumor reaction, independent of the initial thermal effects (6). In addition, the hyperthermia-induced inhibition of angiogenesis may contribute to the anti-tumor reaction.

Angiogenesis, an essential component of pathological and physiological processes, is the formation of new blood vessels from existing vasculature. The predominant stimulator of angiogenesis is vascular endothelial growth factor (VEGF)-A (7). In the majority of cancer types, under pathological conditions, VEGF is secreted by tumor cells and promotes the formation of new blood vessels by acting on the endothelial cells of existing vessels (7,8). The growth and malignant dissemination of solid tumors is dependent on pathological angiogenesis (9). VEGF binds to two associated receptors, VEGF receptor 1 (Flt-1) and 2 (Flk-1 or KDR) (10). Inhibition of VEGF or its signaling via these receptors is a promising strategy to block angiogenesis and the subsequent tumor growth and metastases (8).

Angiogenesis has been reported to be blocked by hyperthermia (11,12). However, the effect of hyperthermic magnetic nanoparticles on the expression of VEGF and VEGF receptors and on angiogenesis has not been elucidated. In the present study, the effect of hyperthermic magnetic nanoparticles on tumor growth, and the expression of VEGF and its receptors were investigated.

## Materials and methods

### Animals

All animal experiments were conducted in accordance with the ‘Guide for the care and use of laboratory animals of the School of Medicine, Tsinghua University’ in Tsinghua University (Beijing, China). Wistar rats with a body weight of ~130 g were purchased from the Institute of Laboratory Animal Sciences, Chinese Academy of Medical Sciences (Beijing, China). This study was approved by the ethics committee of Tsinghua University (Beijing, China).

### Tumor inoculation and treatment

The Walker-256 tumor cells were obtained from the Institute of Materia Medica, Chinese Academy of Medical Sciences (Beijing, China). The rats were injected in the right flanks with 2×10^6^ Walker-256 tumor cells, obtained from an ascitic Walker-256 tumor-bearing rat. Following inoculation, the diameter of the tumors at 8 days ranged between 0.5 and 0.8 cm. Saline or 62.5 mg/ml magnetic nanoparticle fluid, with a volume at 50% of each tumor, was intratumorally injected, as described previously (13). Magnetic Fe_3_O_4_ nanoparticles with a mean diameter of 20 nm were prepared, as previously described (14). The rats were randomly allocated to 5 groups of 24 rats each, including rats injected with saline without magnetic field treatment (NS group), rats injected with magnetic nanoparticle fluid without magnetic field treatment (MF group) and rats injected with magnetic nanoparticle fluid with magnetic field treatment performed once (MFH1 group), twice (MFH2 group) or three times (MFH3 group). The animals were treated with an alternating current magnetic field at a frequency of 180 kHz, 55 GS for 30 min, and the temperatures inside the tumors were monitored with a temperature probe and manually adjusted to 50–55°C, similar to previously described (13). In the MFH2 and MFH3 groups, the subsequent treatment was performed 24 h after the previous treatment.

### Tumor volume measurement

The size of the tumors was measured every 2 days with a caliper. The tumor volume was then calculated with the following formula: Tumor volume = 0.5 × (length × width^2^).

### Immunohistochemistry

For the immunohistochemical staining, the tumors were resected and fixed in a 10% formalin solution 4 days after the hyperthermia treatment. The tumor tissues were sectioned to a 5-μm thickness and fixed to slides. The slides were deparaffinized and incubated with 10% normal serum for 30 min to block background staining. The slides were then incubated for 60 min at 37°C with a rabbit anti-VEGF polyclonal antibody (Santa Cruz Biotechnology, Inc., Santa Cruz, CA, USA) or a rabbit anti-cluster of differentiation (CD)34 polyclonal antibody (Boster Biological Technology, Ltd., Wuhan, China), and subsequently incubated with horseradish peroxidase-conjugated secondary antibodies. Each step was followed by washing with phosphate-buffered saline three times. Peroxidase activity was visualized by treatment with 0.02% diaminobenzidine tetrahydrochloride solution containing 0.005% hydrogen peroxide at room temperature for 5–10 min. The sections were also counterstained with hematoxylin and eosin. Iron in the magnetic nanoparticles was stained with prussian blue. Protein quantification was performed by Imagepro-plus 6.0 software (Media Cybernetics, Rockville, MD, USA), and the intensity values were normalized to the background.

### Reverse transcription polymerase chain reaction (RT-PCR)

Total RNA was extracted using TRIzol (Invitrogen, Carlsbad, CA, USA), as described by the manufacturer. mRNA was reverse transcribed with RevertAid (MBI Fermentas, Inc., Burlington, ON, Canada) at 42°C for 60 min, and the resulting cDNA was subjected to PCR (94°C for 1 min followed by 20–25 cycles at 94°C for 30 sec, 60°C for 30 sec, 68°C for 1 min and an extension for 10 min at 68°C). The PCR products were separated on 1.0% agarose gels and visualized with ethidium bromide. The forward (F) and reverse (R) primer pairs are listed (5′ to 3′) as follows: VEGF-F, CCTGGTGGACATCTTCCAGGAGTACC and VEGF-R, GAAGCTCATCTCTCCTATGTGCTGGC; Flt-1-F, CGGGATCCAAGGGACTCTACACTTGTC and Flt-1-R, GGAATTCCCGAATAGCGAGCAGATTT; Flk-1-F, CATTGTGTCCTGCATCCGGGATAACCT and Flk-1-R, TGTACACGATGCCATGCTCGTCACTGA; 18S-RNA-F, GCCCGAAGCGTTTACTTTGAA and 18S-RNA-R, GGTGAGGTTTCCCGTGTTGA. The quantification of gene expression was performed by Imagepro-plus 6.0 software, and the intensity values were normalized to 18S RNA.

### Statistical analysis

All experiments were performed at least three times and the representative results are shown. Results are expressed as the mean ± standard deviation. The differences between groups were examined for statistical significance using Student’s t-test, except where indicated. Survival rate was assessed using the Kaplan-Meier method and log-rank test. P≤0.05 was considered to indicate a statistically significant difference.

## Results

### Intratumoral administration of MFH induces a high temperature inside Walker-256 tumors

**Figure 1:**
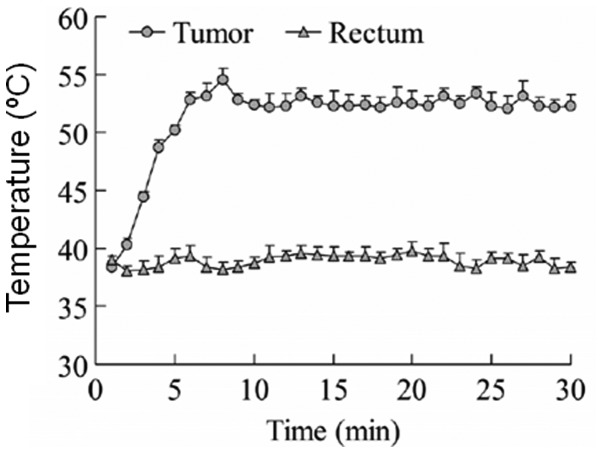
Intratumoral administration of magnetic fluid hyperthermia (MFH) induces a high temperature in the tumors. Rat breast carcinomas were induced by the inoculation of Walker-256 cells in the right flanks of the rats. Tumor diameters reached 0.5–0.8 cm at day 8 following tumor cell inoculation. MFH was intratumorally injected and tumors were treated with an alternating current (AC) magnetic field. The temperature inside the tumor was monitored with a temperature probe once every 2 min following hyperthermia treatment. The temperature inside the rectum was correspondingly monitored as the control.

First, the rat breast carcinoma models were established by inoculation of Walker-256 tumor cells in the right flanks of the rats. When the tumor reached 0.5–0.8 cm in diameter, hyperthermia treatment was performed by the intratumoral administration of MFH, and the temperature in the tumors were measured to determine whether a high temperature had been induced. Fig. 1 shows the mean temperature inside the tumor tissues after hyperthermia treatment, and the temperature inside the rectum as a control. The temperature inside the tumor tissues was increased to 52.5°C rapidly with the MFH treatment, and was maintained within ±2.5°C for the remainder of the treatment. In the controls, the rectal temperature remained normal following the alternating magnetic field treatment. These results indicate that a high temperature was induced successfully in the tumor tissues by MFH.

### MFH treatment using magnetic nanoparticles inhibits tumor growth

**Figure 2:**
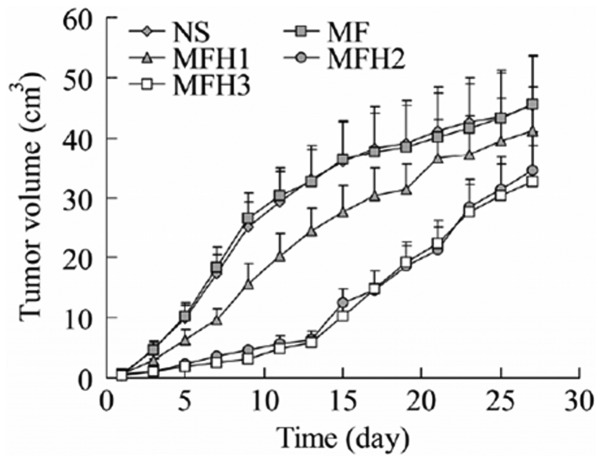
Magnetic fluid hyperthermia (MFH) inhibits tumor growth. Rat breast tumors were induced and treated. The tumor sizes were measured at the indicated time-points. NS, rats injected with saline without magnetic field treatment group; MF, rats injected with magnetic nanoparticle fluid without magnetic field treatment group; MFH1, rats injected with magnetic nanoparticle fluid with magnetic field treatment performed once group; MFH2, rats injected with magnetic nanoparticle fluid with magnetic field treatment performed twice group; MFH3, rats injected with magnetic nanoparticle fluid with magnetic field treatment performed three times group.

MFH has been reported to be feasible for tumor therapy in several cancer types (15–20). In the present study, following the induction of a high temperature in the tumor tissues, attention was paid to the effect of hyperthermia on tumor growth. As shown in Fig. 2, the treatment of tumors with MFH downregulated tumor growth. When compared with the rats injected with saline without the magnetic field treatment (NS group), the rats injected with the magnetic nanoparticle fluid without magnetic field treatment (MF group) had extremely similar tumor growth profiles. These results indicated that the magnetic nanoparticle fluid itself did not affect tumor growth. The rats injected with the magnetic nanoparticle fluid with the magnetic field treatment performed once (MFH1 group) had significantly reduced tumor growth compared with the rats in the NS or MF groups. The rats injected with the magnetic nanoparticle fluid with the magnetic field treatment performed twice (MFH2 group) or three times (MFH3 group) had dramatically reduced tumor growth. The inhibition of tumor growth induced by the MFH treatment lasted for ~2 weeks. The speed of the tumor growth in the MFH2 and MFH3 groups was gradually recovered to that of the NS or MF groups two weeks later.

### MFH treatment promotes survival of tumor-bearing rats

**Figure 3:**
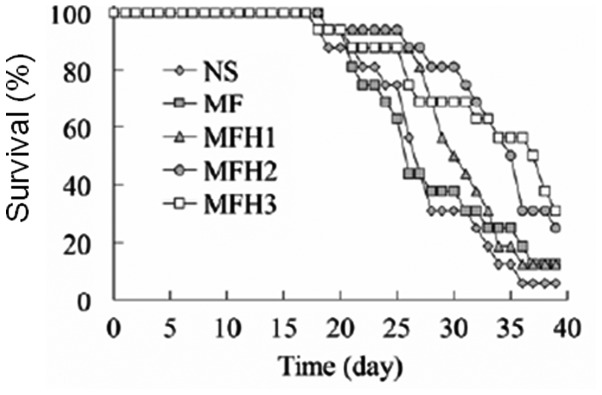
Magnetic fluid hyperthermia (MFH) promotes survival of tumor-bearing rats. The survival curve of rats with or without hyperthermia treatment. Rat breast tumors were induced and treated. The rat survival rate was monitored once every day. NS, rats injected with saline without magnetic field treatment group; MF, rats injected with magnetic nanoparticle fluid without magnetic field treatment group; MFH1, rats injected with magnetic nanoparticle fluid with magnetic field treatment performed once group; MFH2, rats injected with magnetic nanoparticle fluid with magnetic field treatment performed twice group; MFH3, rats injected with magnetic nanoparticle fluid with magnetic field treatment performed three times group.

As it was found that MFH treatment inhibited tumor growth *in vivo*, attention was paid to the effect of MFH on the survival rate of the tumor-bearing rats. The results in Fig. 3 show that consistent with the effect of the MFH treatment on tumor growth, the rats injected with the magnetic nanoparticle fluid with the magnetic field treatment performed twice (MFH2 group) or three times (MFH3 group) had a significantly increased survival rate compared with the rats injected with saline (NS group) or with the magnetic nanoparticle fluid without the magnetic field treatment (MF group) (P<0.05).

### VEGF expression is reduced by hyperthermia treatment

**Figure 4:**
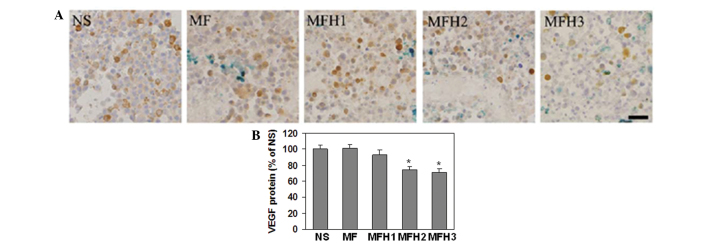
Magnetic fluid hyperthermia (MFH) downregulates the protein expression of vascular endothelial growth factor (VEGF). (A) Representative images of VEGF immunostaining. Rat breast tumors were induced and treated. The tumors were resected and VEGF expression was measured by immunohistochemistry. (B) Quantification of VEGF protein in (A). ^**^P<0.05 vs. NS group. The black bar represents 10 μM. NS, rats injected with saline without magnetic field treatment group; MF, rats injected with magnetic nanoparticle fluid without magnetic field treatment group; MFH1, rats injected with magnetic nanoparticle fluid with magnetic field treatment performed once group; MFH2, rats injected with magnetic nanoparticle fluid with magnetic field treatment performed twice group; MFH3, rats injected with magnetic nanoparticle fluid with magnetic field treatment performed three times group.

Hyperthermia has been reported to inhibit tumor growth by the downregulation of angiogenesis (11,12). To confirm whether angiogenesis was affected in the present rat breast carcinoma model, the VEGF protein in the tumor tissues 4 days after MFH treatment was detected by immunohistochemistry. As shown in Fig. 4A and B, the VEGF protein level in the MFH2 or MFH3 groups was significantly reduced by the MFH treatment compared with that in the NS or MF groups. These results indicate that hyperthermia treatment may affect angiogenesis by the downregulation of VEGF expression.

### MFH downregulates the gene expression of VEGF and its receptors, Flt-1 and Flk-1

**Figure 5:**
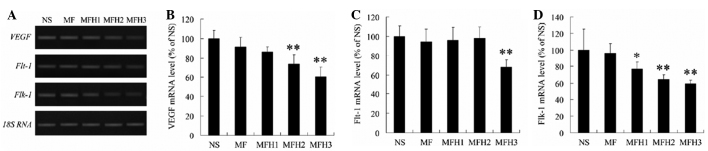
Magnetic fluid hyperthermia (MFH) downregulates the mRNA level of vascular endothelial growth factor (VEGF), VEGF receptor 1 (Flt-1) and 2 (Flk-1). Rat breast tumors were induced and treated. Tumors were resected and the gene expression of VEGF, Flt-1 and FLk1 was detected by reverse transcription polymerase chain reaction (RT-PCR) (A). Gene expression. Amplification of the 18S RNA gene was used as loading controls. (B–D) Densitometric analysis of the qPCR results in (A). ^*^P<0.05 and ^**^P<0.01 versus NS group. NS, rats injected with saline without magnetic field treatment group; MF, rats injected with magnetic nanoparticle fluid without magnetic field treatment group; MFH1, rats injected with magnetic nanoparticle fluid with magnetic field treatment performed once group; MFH2, rats injected with magnetic nanoparticle fluid with magnetic field treatment performed twice group; MFH3, rats injected with magnetic nanoparticle fluid with magnetic field treatment performed three times group.

To further confirm the effect of the MFH treatment on VEGF expression, the gene expression of VEGF and its receptors, Flt-1 and Flk-1, was measured by RT-PCR, and 18S RNA was amplified as the internal control. As shown in Fig. 5A and B, the VEGF mRNA level in the MFH2 or MFH3 groups was significantly reduced by the MFH treatment compared with that in the NS or MF groups, which is consistent with the protein expression of VEGF affected by the MFH treatment. Similarly, the mRNA level of Flt-1 was significantly reduced in the MFH3 group (Fig. 5A and C), and the mRNA level of Flk-1 was slightly reduced in the MFH1 group and markedly downregulated in the MFH2 and MFH3 groups (Fig. 5A and D). These results also indicate that MFH treatment may inhibit angiogenesis by downregulation of VEGF and its receptors, Flt-1 and Flk-1.

### MFH treatment significantly decreases the microvessel density

**Figure 6:**
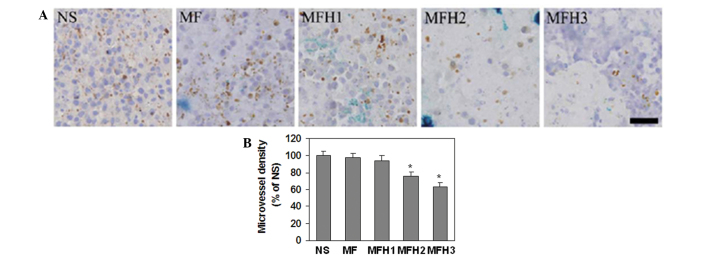
Magnetic fluid hyperthermia (MFH) decreases microvessel density. (A) Representative images of cluster of differentiation (CD)34 immunostaining in the sections of tumors. Rat breast tumors were induced and treated. Tumors were resected and the protein expression of CD34 was detected by immunohistochemistry. The black bar represents 10 μM. (B) Quantification of microvessel density corresponding to (A) using CD34 as a microvessel marker. ^*^P<0.05 vs. NS group. NS, rats injected with saline without magnetic field treatment group; MF, rats injected with magnetic nanoparticle fluid without magnetic field treatment group; MFH1, rats injected with magnetic nanoparticle fluid with magnetic field treatment performed once group; MFH2, rats injected with magnetic nanoparticle fluid with magnetic field treatment performed twice group; MFH3, rats injected with magnetic nanoparticle fluid with magnetic field treatment performed three times group.

CD34 has been reported to be a marker for microvessel density (21–22). Therefore, the microvessel density was detected using immunohistochemistry with the anti-CD34 antibody (Fig. 6), as described previously (23). As expected, the microvessel density in the MFH2 and MFH3 groups was significantly reduced by the MFH treatment compared with that in the NS and MF groups (Fig. 6). The microvessel density in the MFH1 group was not significantly different compared with that in the NS or MF groups (Fig. 6). These results further demonstrated that the MFH treatment with magnetic nanoparticles inhibited angiogenesis.

## Discussion

Similar to the results of a previous study, which showed that hyperthermia treatment with magnetic nanoparticle fluid at ~54°C significantly inhibited tumor growth in a rat tumor model induced by implantation of MatLyLu-cells into the prostates of rats (13), the present study also observed that hyperthermia treatment at 52.5±2.5°C reduced Walker-256 tumor growth. However, following relatively long-term observation, it was found that 2 weeks after hyperthermia treatment, the tumor growth recovered partly to the levels in the control groups. In addition, it was also found that performing the MFH treatment once did not inhibit tumor growth significantly, but that repeating the MFH treatment two or three times inhibited tumor growth and also promoted the survival of the tumor-bearing rats. These results indicate that in order to properly control tumor growth, repeated and lasting MFH treatment is required.

VEGF and its downstream signaling play a significant role in angiogenesis and tumor progression (7,8). Hyperthermia has been reported to inhibit the expression of VEGF and its receptors, which downregulates angiogenesis and thus facilitates the inhibition of tumor growth. For example, hyperthermia at 42°C was shown to suppress the gene and protein expression of VEGF in human fibrosarcoma HT-1080 cells, and the level of VEGF in sera from cancer patients was significantly diminished 2–3 weeks after treatment with whole-body hyperthermia at 42°C (24). In addition, it was reported that heat exposures between 41 and 45°C also directly inhibited angiogenesis in mice (11,12). In the present study, it was shown that hyperthermia treatment at 52.5±2.5°C inhibited the expression of VEGF, Flt-1 and Flk-1 and inhibited angiogenesis. As magnetic nanoparticles alone did not affect the expression of VEGF, Flt-1 and Flk-1 and angiogenesis, it was concluded that the effect of magnetic nanoparticle-induced hyperthermia on angiogenesis is similar to that induced by other heat exposures.

Taken together, the present study results showed that hyperthermia treatment at 52.5±2.5°C using magnetic nanoparticles inhibited tumor growth, promoted the survival of the tumor-bearing rats and inhibited angiogenesis potentially by the downregulation of the expression of VEGF and its receptors, including Flt-1 and Flk-1. These results indicate that the hyperthermia-induced inhibition of VEGF and its receptors may be involved in tumor thermotherapy.
